# STRspy2.0: Unlocking the Potential of Long Reads for Forensic DNA Profiling

**DOI:** 10.3390/ijms27041889

**Published:** 2026-02-16

**Authors:** Courtney L. Hall, Rupesh K. Kesharwani, Katherine E. McBroom Henson, Bupe Kapema, Nicole R. Phillips, Fritz J. Sedlazeck, Roxanne R. Zascavage

**Affiliations:** 1 Department of Biomedical Engineering, Johns Hopkins University, 3400 N Charles St., Baltimore, MD 21201, USA; chall106@jh.edu; 2 Department of Neurology, The University of Texas Health Science Center at Houston, 7000 Fannin St., Houston, TX 77030, USA; fritz.sedlazeck@bcm.edu; 3 Human Genome Sequencing Center, Baylor College of Medicine, One Baylor Plaza, Houston, TX 77030, USA; 4 Department of Microbiology, Immunology & Genetics, University of North Texas Health Science Center, 3500 Camp Bowie Blvd., Fort Worth, TX 76107, USA; katherinemcbroom@my.unthsc.edu (K.E.M.H.); nicole.phillips@unthsc.edu (N.R.P.); roxanne.zascavage@unthsc.edu (R.R.Z.); 5 Department of Computer Science, Rice University, 6100 Main St., Houston, TX 77030, USA

**Keywords:** DNA sequencing, human identification, short tandem repeats, nanopore sequencing, bioinformatics, STRspy

## Abstract

Forensic human identification relies on length-based differences in short tandem repeats (STRs) across autosomal and Y chromosomes, which require separate reactions and provide limited resolution. While next-generation sequencing offers greater discriminatory power, most platforms are expensive and restricted to traditional lab settings. Nanopore sequencing has the potential to change this with the real-time, portable MinION sequencer. However, forensic-specific tools that generate STR profiles compatible with established length-based databases are lacking. To address this, we developed STRspy2.0, which simultaneously profiles autosomal and Y-STRs using nanopore reads. STRspy2.0 produced accurate profiles for 54 multiplexed control libraries and 41 mock casework samples (blood, swab, bone), achieving overall F1-scores of 100% and 99.75%, respectively. It maintains compatibility with existing forensic databases while providing higher resolution than traditional profiles. Our updated method and comprehensive database, along with the MinION’s small size and price, make sequence-based STR profiling more accessible to forensic laboratories and resource-limited settings.

## 1. Introduction

Forensic DNA examinations harness short tandem repeats (STRs) across both autosomal and Y chromosomes for human identification in routine casework [1,2,3]. Traditional STR profiling involves PCR amplification and fluorescent labeling of target loci that are then separated and detected with capillary electrophoresis (CE) [4]. Differences in repeat length across autosomal STR panels achieve enough statistical power to differentiate between individuals, link DNA from a crime scene to a known source, and confirm familial relationships [5,6]. When male DNA is present, Y-STRs are often profiled using the same methods as autosomal STRs but require separate sample normalization, PCR, and CE [7]. This consumes often limited DNA evidence and creates a backlog by prolonging the period in which a case is being processed. Although powerful and reliable, CE profiles can only provide length-based resolution for up to 35 STR loci at a time [8], highlighting the need for more efficient and comprehensive approaches in forensics.

The high sample throughput and enhanced multiplexing capabilities of next-generation sequencing (NGS) platforms enable more powerful STR profiles to be generated in less time than conventional typing techniques [9,10]. Short-read Illumina sequencing platforms have been instrumental to our understanding of allelic diversity, uncovering nucleotide-level variation within and around STRs not observed in length-based CE profiles [10]. These data revealed population-specific flanking region single nucleotide polymorphisms (SNPs) and isoalleles, which have the same length but different underlying sequences. NGS has identified more than twice as many sequence-based alleles compared to CE at some STRs [10]. This increase in resolution across established forensic panels has proven critical for challenging casework, such as degraded samples, complex kinship analyses, and mixture deconvolution.

Integrating NGS data into length-based STR databases challenges existing forensic nomenclature and data interpretation standards [11]. These limitations have motivated efforts to develop and validate forensic NGS workflows and data analysis tools [12,13,14]. The Illumina MiSeq FGx Forensic Genomics System, the first approved for upload to the National DNA Indexing System (NDIS) database, remains the most well-established NGS platform for forensic STR analysis [15]. However, the high upfront cost (>$100,000) has limited widespread adoption of sequence-based STR typing. Most forensic laboratories cannot allocate resources to purchase and validate the MiSeq FGx while maintaining conventional STR typing workflows. As a result, analysts are often forced to outsource NGS testing, increasing casework backlogs and delaying the generation of investigative leads.

With a startup cost of just $3000, nanopore sequencing on the portable MinION device from Oxford Nanopore Technologies (ONT) is an affordable alternative for uncovering hidden variation in current STR profiles. Unlike the benchtop MiSeq FGx, which confines forensic analysis to well-equipped labs with multi-day workflows, the pocket-sized MinION could support real-time STR profiling at crime scenes, police stations, or remote locations—cutting turnaround times from days to hours and helping ease national backlogs [16]. Although the MinION could revolutionize forensics, nanopore STR profiling is challenging due to the lack of computational methods that can generate accurate sequence-based STR profiles compatible with established length-based forensic databases using ONT reads.

We previously developed STRspy, a streamlined bioinformatic method capable of producing accurate sequence- and length-based forensic profiles across an entire panel of autosomal STRs using ONT reads and has been explored in various forensic research projects [17,18,19,20]. However, STRspy could only profile autosomal loci and relied on a limited STR allele database to do so. This database was built on historical CE-derived allele nomenclature, which creates inconsistencies when applied to NGS data, and lacked the allelic diversity needed to accurately profile different populations [10,11,21,22]. Expanding the STR allele database to include additional autosomal targets and alleles was time-consuming, and required more advanced computational skills.

**Figure 1:**
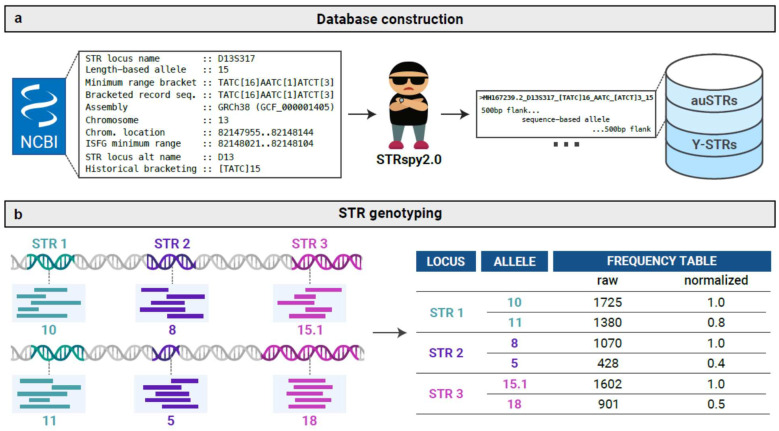
Schematic overview of STRspy2.0 workflow. (**a**) STRspy2.0 relies on an STR allele database to generate profiles consistent with established length-based forensic nomenclature. Users can use the STRspy2.0 database or build their own from GenBank records. When GenBank records are provided, STRspy2.0 automatically generates database entries using the locus name, reference chromosome, repeat location, repeat sequence, and flanking sequence variants, then adds 500 bp flanks from hg38 to each allele. (**b**) During data analysis, STRspy2.0 aligns reads to the human reference genome, extracts those overlapping STR loci, and realigns them to the STR allele database. Each allele is ranked by normalized read count to determine the genotype at each locus. Partially created in Biorender, Mcbroom, K. (2025) Https://Biorender.Com/5alv8be.

To address these limitations, we expanded our method to support simultaneous profiling of all loci and alleles reported in the common autosomal and Y-STR subdivisions of the STR Sequencing Project (STRSeq) [23], in accordance with current recommendations from the DNA Commission of the International Society of Forensic Genetics (ISFG) [11]. While STRspy2.0 maintains the original STRspy framework, it now allows for Y-STR profiling, improved command-line arguments, an updated STR allele database, and a script that can automatically create database entries from GenBank records (Figure 1).

We first validated STRspy2.0 using four high-quality reference materials sequenced on the MinION. STRspy2.0 achieved perfect concordance with manufacturer-validated profiles across 22 autosomal and 23 Y-STRs based on length. We then benchmarked STRspy2.0 using DNA from 41 human tissues processed in routine forensic casework (blood, buccal swabs, bone). These casework-relevant samples demonstrate that STRspy2.0 can generate accurate ONT profiles from true unknowns and highlight forensic-specific challenges associated with implementing NGS in a system that was established based on CE. Collectively, this shows STRspy2.0 can use ONT sequencing data from diverse human tissues and populations to produce accurate profiles across one of the largest autosomal and Y-STR amplification panels available, therefore decreasing cost and increasing the forensic potential of the MinION device in future applications.

## 2. Results

### 2.1. Control DNA Multiplexes

NGS platforms provide higher sample throughput and enhanced multiplex capabilities over conventional CE typing techniques. In addition to 22 autosomal STRs, the PowerSeq 46GY System amplifies 23 STRs on the Y chromosome. To harness all genetic information produced using commercial NGS amplification kits, we expanded the STRspy2.0 framework to support simultaneous profiling of autosomal and Y-STRs. These updates were first assessed using 4 standard reference materials for forensic STR profiling (NIST A, NIST B, NIST C, 2800 M). After PowerSeq amplification, stock solutions of barcoded ONT libraries were pooled and sequenced in sets of 12, 18, and 24 samples on the MinION device. Basecalled reads from the 54 datasets were then profiled with STRspy2.0 using the default settings. Briefly, STRspy2.0 maps all reads to the human reference genome, extracts primary alignments that span target STR loci, and remaps them to our updated allele database. Read counts are then normalized to the allele with the highest coverage and these normalized values are used to predict the autosomal genotype or Y haplotype at each locus. STRspy2.0 reports read mapping and coverage statistics as well as bracketed repeat motifs and length-based allele designations consistent with conventional forensic nomenclature. This naming system is both unique and specific to forensics, making it difficult to extract CE alleles from NGS data *de novo*. To overcome this challenge, each sequence-based allele in the STRspy2.0 database is labeled with the bracketed repeat motif and length-based designations, as reported in NCBI [23]. STRspy2.0 can therefore provide nucleotide-level information alongside CODIS-compatible profiles without additional processing.

**Figure 2:**
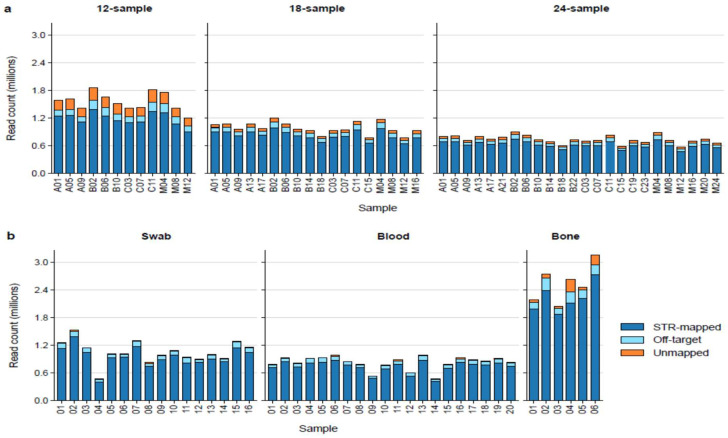
Read mapping statistics for MinION sequencing runs. Bar plots showing the proportion of passed reads that were STR-mapped (on-target), off-target, and unmapped for the (**a**), control multiplexes and (**b**), casework-relevant samples. Each plot shows samples loaded onto a single MinION flow cell. Libraries in the control multiplex are labeled according to sample letter and barcode. A (NIST A), B (NIST B), C (NIST C), M (2800 M).

We first evaluated how multiplex size impacts sequencing coverage and STRspy2.0 processing time. As expected, increasing the number of samples per flow cell reduced the number of reads generated per sample (Figure 2a, Supplementary Table S1). Basecalled reads passing QC ranged from 573,306 (24 sample multiplex) to 1,849,407 (12 sample multiplex), with an average of 998,906 per sample. Despite differences in total read depth, on-target mapping rates remained consistent across all multiplexes, ranging from 87.0% to 91.3% (mean ± SD: 89.7% ± 1.00%). Locus-level coverage was also well-balanced, with uniform representation of all targeted regions and no evidence of systematic dropout (Supplementary Table S2). These results demonstrate that STRspy2.0 maintains reliable targeting and sufficient coverage across a wide range of multiplex conditions.

To evaluate how coverage impacts profiling speed, we measured STRspy2.0 runtime using a single CPU thread and 100 GB of RAM per sample. Starting with unaligned FASTQ files, STRspy2.0 generated complete STR profiles for the 54 control datasets in ~96 h, averaging one hour and 47 min per sample. When using aligned BAM files as input, runtime was reduced to under 80 min for each control in the multiplexing experiment. As expected, runtime also decreased with increasing multiplex size due to lower coverage across target loci. This trend is consistent with previous findings that STRspy runtime scales with sequencing depth and can be further accelerated through multithreading [17]. Together, our results show that STRspy2.0 offers a practical and scalable solution for high-throughput forensic applications even with limited computing resources.

We then evaluated the accuracy of STRspy2.0 profiles for the 22 autosomal and 23 Y-STRs in the multiplexed datasets. STRspy2.0 predicted the correct length-based allele designations for all multiplexed samples, resulting in a recall, precision, and F1-score of 100% (Table 1). These results demonstrate that STRspy2.0 can generate accurate and reproducible profiles using ONT data from the largest sample multiplex assessed to date.

**Figure 3:**
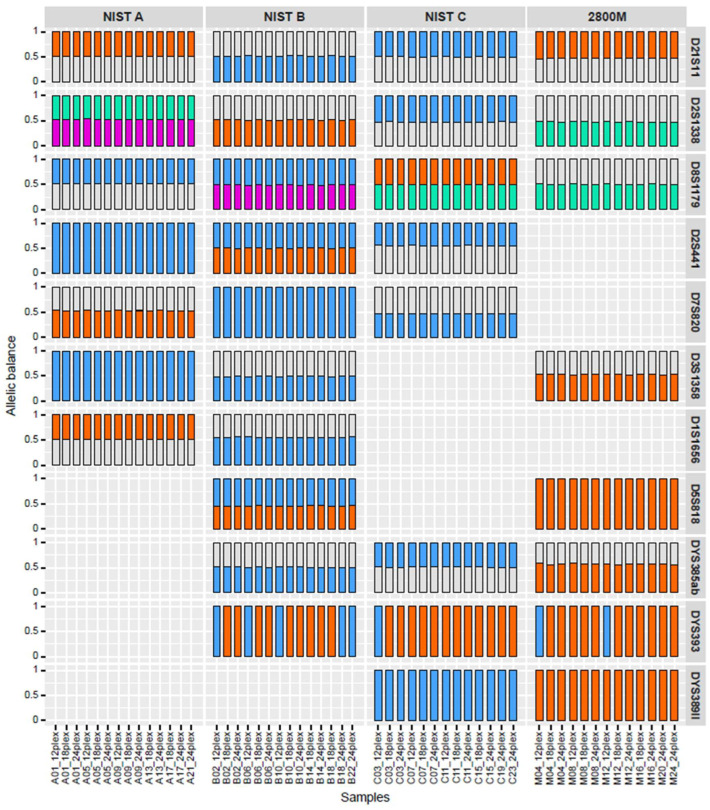
STRspy2.0 can resolve isoalleles within and between individuals. Allelic balance for autosomal and Y-STR isoalleles detected across control samples in the multiplexing experiment. Each colored bar represents a distinct sequence-based isoallele. Isoallele pairs are shown as blue/orange (pair 1) and green/pink (pair 2) for each locus. Heterozygous alleles that are not in the isoallele pair are colored grey, and samples without isoalleles at a given locus are not shown. Libraries are labeled according to sample letter, barcode, and multiplex size. A (NIST A), B (NIST B), C (NIST C), and M (2800 M).

In addition to supporting larger multiplexes, NGS can be used to differentiate between isoalleles, which have the same length in traditional CE profiles but different underlying sequences. Sequence-based profiles can therefore achieve a higher power of discrimination across established panels of forensic STRs than CE. To assess STRspy2.0’s ability to detect isoalleles, we compared its sequence-based allele predictions to manufacturer-validated NGS profiles across the control datasets. Despite differences in coverage, STRspy2.0 resolved all autosomal isoalleles within and between control DNA samples (Figure 3, Supplementary Table S3). At D2S441 in NIST B, for example, the number of reads supporting each sequence-based allele ([TCTA]11; [TCTA]9 TCTG TCTA) for the length-based 11 homozygotes ranged from 3518 to 4393 in the 12-sample multiplex, 2644 to 4015 in the 18-sample multiplex, and 1972 to 2862 in the 24-sample multiplex. Nevertheless, STRspy2.0 reported the correct length- and sequence-based designations, highlighting the robustness of our method.

STRspy2.0 was also able to resolve most Y-STR isoalleles across the control multiplexes (Figure 3). At DYS389II, it correctly identified the bracketed repeat motifs for the length-based 31 isoalleles in NIST C ([TAGA]9 [CAGA]3 N48 [TAGA]13 [CAGA]6) and Promega 2800 M ([TAGA]11 [CAGA]3 N48 [TAGA]13 [CAGA]4). STRspy2.0 was even able to distinguish between isoalleles at homopolymer-rich Y loci, such as DYS385ab. At DYS393, all 40 male replicates (NIST B, NIST C, and 2800 M) were assigned the correct allele length (13) by STRspy2.0. However, only eight matched the manufacturer-validated sequence motif [AGAT]13. The other 32 predictions were miscalled as CGAT [AGAT]12, differing from the true allele by a single base (A vs. C) in one of the outer repeats.

Overall, STRspy2.0 was able to successfully resolve 414 of 446 isoalleles (92.8%) across eight autosomal and three Y-loci in the control DNA dataset with miscalls occurring in only one Y-locus (Figure 3). Our updated method can therefore reveal nucleotide-level variation in ONT reads with high accuracy, producing more powerful autosomal and Y-STR profiles than conventional CE approaches with a single PCR reaction and sequencing run.

### 2.2. Casework-Relevant Samples

The previous multiplexing control experiment was conducted with high-quality reference materials; however, DNA evidence encountered in routine forensic laboratories is often less pure and more degraded than these manufacturer-extracted controls. To demonstrate that our methods are suitable for casework-relevant biological material, we sequenced and profiled STR amplicons from 19 blood, 16 buccal swab, and 6 bone samples using STRspy2.0 with the default settings. As seen with the controls, read coverage decreased as more samples were loaded per MinION flow cell (Figure 2b). Nevertheless, our method produced a high percentage of on-target reads and sufficient depth of coverage across all autosomal and Y-STRs in blood, swab, and even bone for profiling with STRspy2.0 (Supplementary Table S4).

STRspy2.0 profile predictions were compared to CE (swab, bone) or NGS (blood) allele designations (Supplementary Table S5). The number of true positives, false positives, and false negatives predicted by STRspy2.0 were used to calculate recall, precision, and F1-score (Table 1). STRspy2.0 correctly identified 1962 of the 1972 autosomal and Y alleles across the casework dataset, achieving an overall F1-score of 99.8% (bone: 100%; blood: 99.9%; swab: 99.5%). The 10 incorrect calls were distributed between blood samples (2) and swab samples (8). In blood samples, we observed 1 false positive out of 167 Y-STR alleles (F1-score: 99.7%) and 1 false negative out of 799 autosomal alleles (F1-score: 99.9%). Swab samples contained more errors overall, with 3 false positives and 1 false negative in 640 autosomal STR calls (F1-score: 99.7%) and 4 false positives in 158 Y-STR calls (F1-score: 98.7%). While most errors could be attributed to common sequencing artifacts or analytical factors, others lacked a clear explanation. A detailed breakdown of the 10 incorrect calls is presented in Table 2.

Skeletal remains, which often contain low quantities of degraded DNA with endogenous contaminants, are challenging to profile with traditional CE approaches. This is evident by the inability to obtain length-based reference profiles for the Y chromosome of our bone samples using CE. Nanopore STR sequencing has not previously been evaluated in bone, with a recent study even concluding that their ONT-specific panel would not be suitable for bone extracts [24]. Still, STRspy2.0 correctly called all autosomal alleles across the bone samples, resulting in recall, precision, and F1-score of 100% (Table 1).

The bone benchmarking presented in Table 1 is limited to length-based autosomal designations with successful CE profiling results (Supplementary Table S5). Due to the inability to generate a CE-based reference for the Y STRs, the accuracy of STRspy2.0 for generating length-based alleles for Y-STRs from bone was not assessed. To cross-validate our results and evaluate the ability for STRspy 2.0 to generate Y-STR profiles from challenging bone samples, two bone extracts (bone02, bone03) were sequenced and profiled using the CODIS-validated workflow for STR profiling on the Illumina MiSeq FGx System. We compared sequence-based allele calls across shared loci in the Illumina and ONT profiles generated using the ForenSeq Universal Analysis Software and STRspy2.0, respectively (Supplementary Table S6). STRspy2.0 showed complete concordance with Illumina profiles for all autosomal and Y-STRs in both bone samples, demonstrating that our method can produce reliable results from challenging forensic samples.

**Figure 4:**
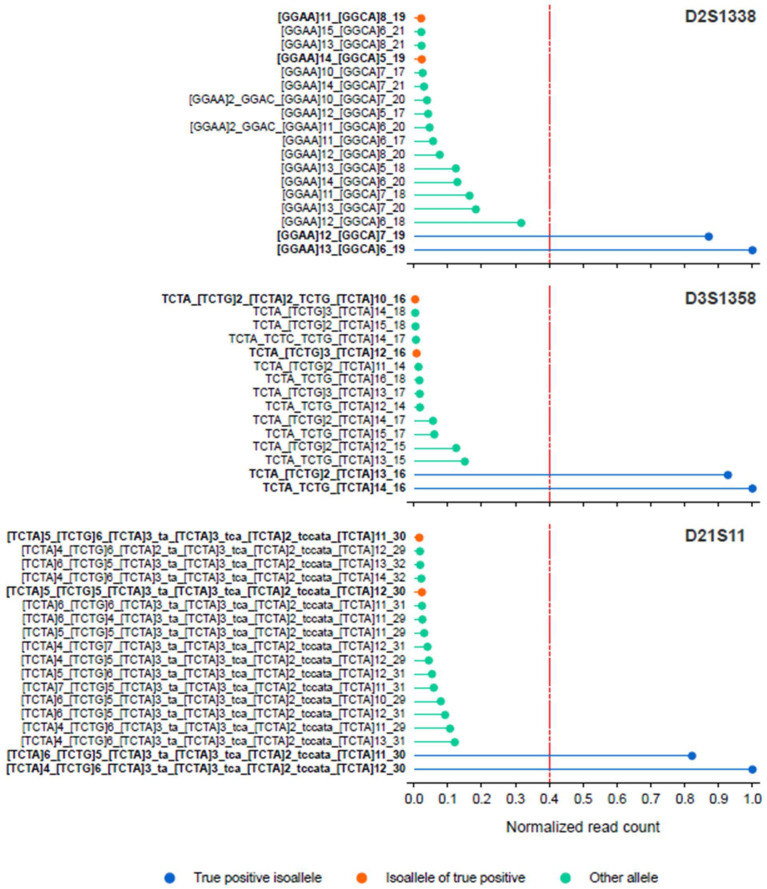
STRspy2.0 can resolve isoalleles in challenging casework-relevant samples. Normalized read counts for sequence-based alleles detected at D2S1338, D3S1358, and D21S11 in one bone sample (bone03). The red dashed line indicates the normalization threshold (0.4) used to detect heterozygous alleles.

We also assessed the ability of STRspy2.0 to resolve isoalleles using ONT sequencing data for the casework samples. We identified three autosomal loci (D2S1338, D3S1358, D21S11) in one of the bone extracts (bone03) that contain isoalleles according to the Illumina profiles. STRspy2.0 was able to distinguish between these length-based homozygotes, correctly calling different sequence-based alleles using the ONT sequencing data (Figure 4). These results further highlight that our method can resolve isoalleles to achieve higher resolution than CE, producing sequence-based profiles consistent with those generated on the extensively validated and much more expensive Illumina MiSeq FGx System.

## 3. Discussion

Here, we present STRspy2.0, an expansion of our original method that now supports simultaneous profiling of autosomal and Y-STRs using ONT sequencing data. Unlike our previous proof of concept, which was limited in scope, STRspy2.0 achieves accurate profiling across diverse human tissues, ranging from high-quality reference materials to challenging casework-relevant samples such as bone. While this study focused on ONT data, the general framework could also be adapted for single-molecule sequencing on PacBio platforms. However, ONT’s portability and low startup cost make it better suited for rapid or field-based forensic analysis than Illumina or PacBio sequencers, which require fixed laboratory infrastructure. We designed STRspy2.0 to be as accessible to forensic laboratories as the MinION sequencer itself. This release features a complete overhaul of the allele database and a new script that automatically generates entries from GenBank, reducing the computational barrier for end users and streamlining future database updates. STRspy2.0 is open source, lightweight, and platform agnostic, allowing forensic laboratories to perform the entire sequencing workflow—from data collection to analysis—on a standard laptop without the need for specialized computing resources.

Long-read, single-molecule sequencing technologies have high error rates in low-complexity regions, making it challenging to accurately characterize STR repeat-length variation [25]. Existing tandem repeat callers, such as TRGT [26] and Straglr [27], can detect simple STR motifs in error-prone reads but cannot handle the compound and complex repeats in core CODIS panels or provide STR allele designations consistent with established forensic databases. In contrast, forensic NGS tools like FDSTools [28] and STRait Razor [29] report STR profiles in the correct format, but were designed for Illumina data and have not been validated on ONT reads. Previous attempts at nanopore-based STR profiling have only achieved partial concordance (90–92%) with CE results and have been limited in both sample diversity and scope [30,31,32,33,34]. STRspy2.0 produces near perfect accuracy across four control DNAs, including 100% concordance for 2800 M (which showed only 73.5% concordance in a recent study [30]), and 41 casework-relevant samples sequenced on the ONT MinION. STRspy2.0 also reports both sequence- and length-based allele designations to reveal nucleotide-level variation while maintaining compatibility with established CODIS databases.

As NGS becomes more widespread in forensics, issues related to nomenclature have emerged as a major challenge [11,21,22]. The traditional forensic naming system was developed based on CE, which assigns allele designations using an internal sizing ladder. Alleles with nucleotide-level differences that do not change fragment size, including isoalleles and flanking region variants, have the same length-based designation. This has created inconsistencies when comparing CE profiles to the more detailed sequence-based profiles [11,21,22]. To address these challenges, the DNA Commission of the International Society for Forensic Genetics (ISFG) outlined recommendations for sequence-based STR nomenclature that leverage the nucleotide-level information in NGS profiles while maintaining compatibility with CE databases [11]. In line with these suggestions, NCBI updated the bracketed motifs and length-based allele designations for all loci in the STRSeq BioProject (e.g., CSF1PO 10 allele: [AGAT]10 vs. [TCTA]_[ATCT]3_[TCTA]2—Supplementary Table S6) but these changes have not been universally adopted across U.S. forensic laboratories. As a result, STR profiles generated in different labs or even on different platforms can be difficult to reconcile, complicating database searches and interlaboratory profile comparisons. The STRspy2.0 database was built on the updated STRSeq records and provides standardized nomenclature aligned with ISFG recommendations, thus helping bridge historic CE-based practices with modern sequence-based forensic profiling.

STRspy2.0 advances forensic genetics by delivering portable, accurate, and sequence-resolved STR profiling. By reporting sequence-based allele information alongside conventional length-based nomenclature, STRspy2.0 provides higher discriminatory power than CE while maintaining compatibility with existing CODIS databases. Coupled with ONT’s portability and affordability, this framework represents a step toward practical, field-deployable forensic sequencing. With continued improvements in nanopore chemistry and community-driven database expansion, STRspy2.0 could make forensic STR sequencing on the MinION a viable alternative to larger, more expensive NGS platforms.

## 4. Methods

### 4.1. Samples

Control DNAs: The multiplexing experiment was conducted using 3 NIST traceable standards and 1 Promega control (female *n* = 1; male *n* = 3) with manufacturer-validated CE and NGS STR profiles. NIST A, B, and C (SRM 2391d) were quantified on the Qubit 2.0 Fluorometer using the Qubit dsDNA BR Assay (Thermo Fisher Scientific (Waltham, MA, USA)) and diluted to 0.1 ng/µL in amplification grade water. The positive control included in the PowerSeq 46GY System (2800 M, Promega, Madison, WI, USA) was prepared and normalized as per manufacturer recommendations. The Qubit 1X dsDNA HS Assay (Thermo Fisher Scientific) was used to confirm the final concentration of all control DNAs before PowerSeq amplification and ONT library preparation.

Blood: Whole blood samples (female: *n* = 10; male: *n* = 9) were selected at random from the PRECISION Pain Research Biobank. DNA was extracted from 100 µL of each sample with the DNeasy Blood & Tissue Kit (Qiagen Sciences, Germantown, MD, USA) spin protocol and normalized using the same methods as the NIST control DNAs prior to amplification for ONT (0.1 ng/µL) and Illumina (0.2 ng/µL) library preparation.

Buccal swabs: DNA was extracted from 16 buccal swab samples (female: *n* = 6; male: *n* = 10) according to the QIAamp DNA Mini Blood Kit (Qiagen) spin protocol with the optional centrifugation step at full speed before elution in 50 µL of buffer AE (Qiagen). DNA extracts were then quantified on the Applied Biosystems 7500 Real-Time PCR System using the Quantifiler Trio DNA Quantification Kit (Thermo Fisher Scientific) as per manufacturer protocol and normalized based on either the small autosomal or Y target for the CE and NGS workflows described below.

Bone: Six human bone samples (female *n* = 1; male *n* = 5) were obtained from the University of North Texas Center for Human Identification (UNTCHI). Samples were extracted using a Demineralization Extraction of Skeletal Remains protocol [35]. They were then quantified and profiled with CE by the UNTCHI Missing Persons Laboratory as a part of previous studies. The four bone extracts below 0.1 ng/µL (bone01, bone04, bone05, bone06) were concentrated in an Eppendorf 5301 Vacufuge System (Eppendorf, Hamburg, Germany) before PowerSeq amplification. The two samples with higher quantification values (bone02, bone03) were normalized based on the small autosomal target for ONT (0.1 ng/µL) and Illumina (0.2 ng/µL) library preparation.

### 4.2. CE Profiles

STR profiles generated using the conventional PCR-CE approach served as the ground truth for the buccal swab and bone samples. Normalized buccal swab extracts were amplified in half reactions using the GlobalFiler and YFiler Plus PCR Amplification Kits (Thermo Fisher Scientific) on the Applied Biosystems GeneAmp PCR System 9700 (Applied Biosystems, Foster City, CA, USA). Autosomal STRs were profiled by the UNTCHI Missing Persons Laboratory with either the AmpFLSTR Identifiler Plus PCR Amplification Kit (bone01, bone02, bone03, Thermo Fisher Scientific) or PowerPlex Fusion 5C System (bone04, bone05, bone06, Promega). All CE data were collected on the Applied Biosystems 3130xl Genetic Analyzer (Thermo Fisher Scientific) and visualized with GeneMapper ID-X Software (v1.7).

### 4.3. Illumina Profiles

Length- and sequence-based STR profiles for the 20 blood samples were generated on the MiSeq FGx Sequencing System (Illumina, San Diego, CA, USA) with the NDIS-approved ForenSeq DNA Signature Prep Kit (Verogen, San Diego, CA, USA). Two bone (bone02, bone03) extracts were also sequenced to demonstrate the correctness of STRspy2.0 repeat motif predictions in biological materials typed via CE. PCR reactions containing 1ng of DNA were prepared with primer mix A (DPMA, Illumina) and amplified on the Eppendorf Mastercycler pro S (Eppendorf) as per manufacturer protocol. After bead-based normalization, the mock casework samples, as well as positive and negative controls, were pooled and diluted in hybridization buffer (HT1, Illumina). The final library was then denatured and loaded into the reagent cartridge for sequencing. Paired-end reads were analyzed with the ForenSeq Universal Data Analysis Software (v2.5.0x).

### 4.4. ONT Profiles

The 22 autosomal and 23 Y-STRs in the PowerSeq 46GY System (Promega) were amplified for ONT sequencing using 0.5 ng of DNA. Amplification was performed with the recommended thermal profile at 30 cycles on the Eppendorf Mastercycler pro S (Eppendorf). STR amplicons were then processed with the QIAquick PCR Purification Kit (Qiagen) according to the microcentrifuge protocol. A 10 µL aliquot of 3M sodium acetate (pH 5.0) was added to all samples before column binding due to the observed change in color of the pH indicator. DNA was eluted in 50 µL of nuclease-free water, resulting in 48 µL of purified amplicons for ONT library preparation.

STR libraries were prepared using the ONT (ONT, Oxford Science Park, Oxford, UK) Ligation Sequencing Kit (SQK-LSK109) with Native Barcoding Expansions 1–12 (EXP-NBD104) and 13–24 (EXP-NBD114) as per the modifications described in Hall et al. [17]. Purified amplicons from one PCR reaction (48 µL) were used as the input for ONT library preparation. Following end-repair and dA-tailing, unique barcodes were ligated onto both amplicon ends in samples to be sequenced together. The multiplex experiment was performed using stock solutions of barcoded samples to eliminate potential variation in library preparation. The four control DNAs were labeled using all 24 barcodes available for the ligation-based workflow at the time of data generation. To ensure that sufficient stock solution was available to sequence and resequence different multiplex combinations if needed, six to eight amplicon libraries were prepared and pooled per barcode. Bead-purified samples were then quantified on the Agilent TapeStation 4200 (Agilent, Santa Clara, CA, USA) with D1000 ScreenTapes and combined according to the concentration of fragments ranging from 175 bp to 475 bp. Pooled barcodes exceeding 65 µL were concentrated in an Eppendorf 5301 Vacufuge System (Eppendorf). After ligation of ONT sequencing adapters, amplicon libraries were purified using magnetic beads with two washes in a short fragment buffer (SFB, ONT). Pooled barcodes were then quantified and diluted in elution buffer (EB, ONT) to 75 ng based on overall concentration before preparing final loading libraries. Prepared sequencing libraries were loaded onto primed MinION vR9.4D flowcells (FLO-MIN106D, ONT) and sequenced on the MinION device (ONT) for 72 h with the MinKNOW control software (v22.12.5).

### 4.5. STRspy2.0

Implementation: STRspy2.0 consists of 3 main steps (Figure 1). Basecalled reads are first aligned to the human reference genome (GRCh38/hg38) using minimap2 (v2.24-r1122) [36]. On-target, STR-mapped reads are extracted and realigned to the user-provided allele database. Allele-mapped reads are then normalized to the sequence-based allele with the highest coverage in a per locus manner and this information is used to predict the autosomal genotype or Y haplotype. A detailed account of each step implemented in the STRspy framework is provided in Hall et al. [17]. Here we focus on new features of STRspy2.0.

Automated database construction: STRspy2.0 reports bracketed repeat motifs and length-based allele designations consistent with conventional CE profiles in established CODIS databases and ISFG recommendations [11], using a curated database containing STR alleles at loci of interest. To streamline database creation, we developed a utility script that automatically extracts and reformats relevant information from user-provided GenBank records (gb). This script builds a table containing the GenBank accession number, locus name, reference chromosome, repeat location, repeat sequence, and flanking variation for each allele. Repeat regions with identical nucleotide sequences and flanking variation (e.g., SNPs, indels) are collapsed to eliminate redundancy. Flanking sequences (500 bp upstream and downstream of each repeat) from GRCh38/hg38 are then added to each allele to improve alignment of shorter nanopore reads. Lastly, sequence-based alleles are output in fasta format with information relating to forensic nomenclature (e.g., bracketed repeat motif, length-based allele designation) stored in the header (see Hall et al. [17] for additional details). We used this utility script to construct a comprehensive STR database from GenBank records in the STRSeq BioProject (accessions: PRJNA380345 and PRJNA380347) at the time of publication. Although all common autosomal and Y-STRs in the STRSeq BioProject are included in our database, only loci amplified in the PowerSeq 46GY System (Promega) were assessed in this study.

Simultaneous reporting of autosomal and Y STRs: STRspy2.0 uses normalized read counts to rank the sequence-based alleles detected at each STR of interest. The balance of autosomal alleles is used to predict whether the locus is homozygous (reports top allele) or heterozygous (reports top two alleles) according to the user-defined normalization threshold. The default cutoff of STRspy2.0 is set to 0.4 based on the benchmarking results presented in Hall et al. [17]. Laboratories should adjust this threshold according to their own internal validation studies. For all Y-STRs except DYS385ab, STRspy2.0 reports the allele with the highest normalized read count. DYS385a and DYS385b represent duplications of DYS385 with identical flanking region sequences that are amplified with the same PCR primer pair [37]. After genome-wide mapping and extraction of locus-specific reads, STRspy merges DYS385a and DYS385b aligned reads and reports the top two alleles exceeding the normalization threshold.

### 4.6. Data Analysis

Raw signal data recorded on the MinION device (fast5) were converted to nucleotide sequences (fastq) using ONT’s GPU-enabled Guppy basecaller (v6.4.8) with the super accurate basecalling model (dna_r9.4.1_450bps_sup.cfg). Guppy was also used to demultiplex and merge reads based on barcode. Merged fastq files were then processed with the STRspy2.0 command line interface.

STRspy2.0 outputs allele designations consistent with the established forensic naming system as well as the raw and normalized read counts supporting the prediction. We assessed concordance between STRspy2.0 predictions and known allele designations, or ground truth profiles, generated using a combination of CODIS-validated CE and NGS methods. Each allele that STRspy2.0 reported in the final profile was categorized as a true positive (TP, correct allele), false positive (FP, incorrect allele), or false negative (FN, missing allele). These counts were used to calculate the precision, recall, and F1-score of our updated method. Precision and recall were determined by dividing the number of true positives by the total alleles in the STRspy2.0 (true positive + false positive) or ground truth (true positive + false negative) profiles, respectively. The overall performance of STRspy2.0 for autosomal and Y-STRs was evaluated based on F1-score (harmonic mean of precision and recall).

We first tested STRspy2.0’s new features and updated database using Components A, B, and C of NIST SRM 2391d and 2800 M for a total of 54 datasets with manufacturer-validated CE and NGS profiles across all PowerSeq loci. Benchmarking for mock casework samples was limited to loci and allele designations (i.e., length- or sequence-based) in available ground truth profiles (Supplementary Table S5). To cross-validate STRspy2.0 sequence-based allele calls for samples with only CE ground truth profiles, we also compared ONT and Illumina profiles for 2 bone samples (bone02, bone03) (Supplementary Table S6).

## Figures and Tables

**Table 1:** STRspy2.0 benchmarking results for control and casework samples. Numbers of correct (true positives, TP), incorrect (false positives, FP), and missed (false negatives, FN) predictions for the control multiplexes (NIST A, NIST B, NIST C, 2800 M) and 41 casework-relevant samples (blood, buccal swab, bone) when compared to length-based reference profiles. Recall, precision, and F1-scores are reported as percentages for autosomal and Y-STRs. Y-STRs in the bone samples were not assessed due to a lack of length-based reference profiles.

	Autosomal STRs						Y-STRs					
Dataset	TP	FP	FN	Recall	Precision	F1-Score	TP	FP	FN	Recall	Precision	F1-Score
Control multiplexes												
12-sample	528	0	0	100%	100%	100%	207	0	0	100%	100%	100%
18-sample	792	0	0	100%	100%	100%	299	0	0	100%	100%	100%
24-sample	1056	0	0	100%	100%	100%	404	0	0	100%	100%	100%
Overall	2376	0	0	100%	100%	100%	910	0	0	100%	100%	100%
Mock casework												
Blood	798	0	1	99.9%	100%	99.9%	166	1	0	100%	99.4%	99.7%
Swab	636	3	1	99.8%	99.53%	99.7%	154	4	0	100%	97.5%	98.7%
Bone	208	0	0	100%	100%	100%	-	-	-	-	-	-
Overall	1642	3	2	99.9%	99.82%	99.9%	320	5	0	100%	98.5%	99.2%

**Table 2:** STRspy2.0 genotyping errors in casework samples. Raw and normalized read counts (raw/norm) for the 10 incorrect allele calls observed in blood and swab profiles. Dashes indicate no allele/homozygote identified and bolded alleles denote discrepancies from the ground truth. Read counts displayed represent STRspy calls for the given allele.

Error Type	Sample	Locus	Ground Truth (Raw/Norm)	STRspy2.0 Calls (Raw/Norm)
False negative	blood14	Penta D	5 **2.2 (0/0)**	5 –
	swab15	vWA	17 **16 (2360/0.2)**	17 –
False positive	swab10	D18S51	16 –	16 **15 (2858/0.5)**
	swab12	D8S1179	15 **12 (26/0.002)**	15 **17 (10,791/0.9)**
	swab15	FGA	25 –	25 **24 (5161/0.4)**
	swab02	DYS385ab	**11 (90/0.1)** –	**12 (937/1.0)** **13 (550/0.6)**
	swab14	DYS458	**15.1 (0/0)**	**15 (10,175/1.0)**
	blood07	DYS390	**23 (2112/0.7)**	**22 (2865/1.0)**
	swab16	DYS448	**19 (323/0.06)**	**20 (5274/1.0)**

## Data Availability

Our pre-built STR database and scripts (v2) can be downloaded from the STRspy GitHub page [https://github.com/unique379r/strspy] (accessed on 5 February 2026).

## Supplementary Materials

The following supporting information can be downloaded at: https://www.mdpi.com/article/10.3390/ijms27041889/s1.
